# Design, characterization and control of thermally-responsive and magnetically-actuated micro-grippers at the air-water interface

**DOI:** 10.1371/journal.pone.0187441

**Published:** 2017-12-13

**Authors:** Federico Ongaro, Stefano Scheggi, Arijit Ghosh, Alper Denasi, David H. Gracias, Sarthak Misra

**Affiliations:** 1 Surgical Robotics Laboratory, Department of Biomechanical Engineering, University of Twente, 7522 NB Enschede, The Netherlands; 2 Department of Materials Science and Engineering, The Johns Hopkins University, Baltimore, MD, 21218, United States of America; 3 Department of Chemical and Biomolecular Engineering, The Johns Hopkins University, Baltimore, MD, 21218, United States of America; 4 Department of Biomedical Engineering, University of Groningen and University Medical Centre Groningen, 9713 GZ Groningen, The Netherlands; The Ohio State University, UNITED STATES

## Abstract

The design and control of untethered microrobotic agents has drawn a lot of attention in recent years. This technology truly possesses the potential to revolutionize the field of minimally invasive surgery and microassembly. However, miniaturization and reliable actuation of micro-fabricated grippers are still challenging at sub-millimeter scale. In this study, we design, manufacture, characterize, and control four similarly-structured semi-rigid thermoresponsive micro-grippers. Furthermore, we develop a closed loop-control algorithm to demonstrate and compare the performance of the said grippers when moving in hard-to-reach and unpredictable environments. Finally, we analyze the grasping characteristics of three of the presented designs. Overall, not only does the study demonstrate motion control in unstructured dynamic environments—at velocities up to 3.4, 2.9, 3.3, and 1 body-lengths/s with 980, 750, 250, and 100 *μ*m-sized grippers, respectively—but it also aims to provide quantitative data and considerations to help a targeted design of magnetically-controlled thin micro-grippers.

## Introduction

Microrobotics has the potential to radically reduce the risk and invasiveness of clinical interventions as biopsies, cytoreductions and endarterectomies, as well as cardiac and ophthalmic surgeries [1–10]. In particular, untethered foldable micro-grippers can significantly augment the capabilities of current tethered medical devices for targeted and personalized therapy [11–14]. Nonetheless, there are hurdles that delay the translation of these technologies to clinical use. These are: (1) further miniaturization, (2) untethered actuation, and (3) precise and robust control. Challenges (1) and (2) are related to the need of these micro-grippers to operate in hard to reach environments such as blood vessels. Furthermore, simultaneously satisfying (1) and (2) is a demanding objective, as active actuation of untethered devices typically requires the use of on-board power sources. The miniaturization of these power sources is not yet compatible with operation inside blood vessels. Hence, micro-grippers have to be capable of wirelessly harnessing power from their surroundings [13–16]. Finally, these micro-grippers, when used in surgeries, will have to navigate with high precision in unstructured environments. Here, an excessive control-error might result in damage to the patient. Consequently, (3) is a fundamental requirement.

A promising option to overcome (1) and (2) is the use of thermally-actuated thin micro-grippers. Not only can these grippers be fabricated using nanometer-precision lithography approaches, but also their modeling and control is facilitated by their relevantly easier magnetization axis—due to the magnetic anisotropy of their thin and flat shape. Specifically, thin semi-rigid self-folding micro-grippers have been demonstrated to be capable of performing *in-vitro* and *in-vivo* biopsies [1, 17]. However, the previous *in-vivo* biopsy used an endoscope to deliver the micro-grippers to the specific site of interest. Thus, the technology suffers from a limitation in that the surgery can only be performed in places that are reachable by catheters. Here, we demonstrate closed loop magnetic motion control to wirelessly navigate the surgical microtools along narrow paths, which would not have been possible to achieve using the previous delivery method with endoscopes. Further, the use of a closed-loop motion control algorithm to address (3) could not only increase the retrieval rate of the micro-grippers, but also endow them with increased accuracy and repeatability [18, 19].

Clearly, performance and overall capabilities are also inherently dependent on the micro-gripper design. There are many prior examples how these properties can widely vary from one design concept to another, with grippers offering different trade-offs of specifications as size, velocity, magnetization and grasp reliability [1–8, 10–14]. However, few of these studies have quantitatively analyzed the consequences on performance of variations in single aspects of these designs, such as changes in form-factor, shape and size.

In this study, we design, manufacture, characterize and control four similarly-manufactured untethered, self-folding micro-grippers. Further, we analyze the consequences of shape and size changes on the motion, grasping, and magnetic properties of our designs. To this end, the motion of these micro-grippers is first performed and analyzed in a static environment. Secondly, a prescribed performance based closed-loop controller is used to steer the micro-grippers in a PacMan™-like scenario. Finally, the exploitation of the self-folding capabilities of these grippers for grasping purposes is investigated.

**Fig 1:**
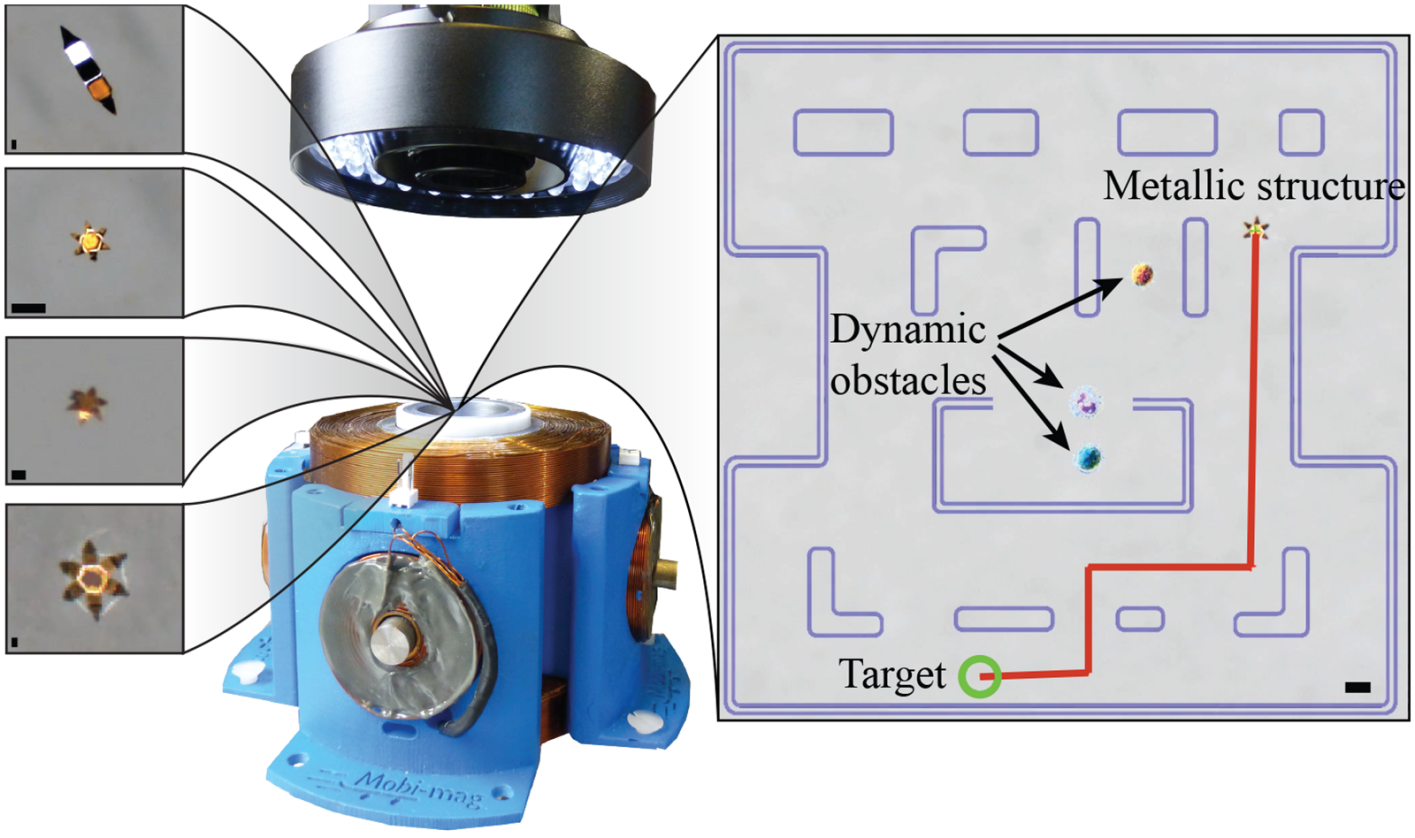
The electromagnetic setup for magnetic motion control consisting of six orthogonally-oriented electromagnetic coils. A maximal current of 3.5 A is used to activate the electromagnetic coils. The setup generates maximum magnetic fields and gradients of 15 mT and 60 mT/m, respectively. *Right inset*: The developed PacMan™-like scenario used for the comparison of the motion control of grippers in dynamic unpredictable environments. *Left insets, from top to bottom*: Microscopy images of: 750 *μ*m two-finger gripper, 100 *μ*m six-finger gripper, 250 *μ*m six-finger gripper, 980 *μ*m six-finger gripper. All scale bars are 100 *μ*m. Unless noted, measurements are reported in the form: mean value ± standard deviation.

We previously demonstrated manipulation of soft micro-grippers in an environment with moving obstacles and using ultrasound imaging [8, 10]. Here, we aim to push such technology towards navigation inside unpredictable micro-channels at considerably smaller length scales. Accordingly, the micro-grippers in this study are miniaturized down to forty times smaller sizes than our previous work. Additionally, differently from our previous work [8, 10], the various micro-grippers are moving in virtual micro-channels. Further, in these micro-mazes, the moving obstacles do not follow a fixed trajectory; instead, the obstacles dynamically plan their trajectory to attack the controlled grippers (Fig 1).

## Materials and methods

### Fabrication of the self-folding micro-grippers

The metallic micro-grippers used in this work are manufactured using conventional multilayer microfabrication techniques and can thus be produced in large numbers. A sacrificial layer of 100 nm of copper is thermally evaporated on a clean Si wafer to facilitate the lift-off of the micro-grippers. UV photolithography is used to define the patterns for the thermal deposition of the differentially stressed bilayer, which consisted of 60 nm chromium and 100 nm gold. This is followed by a second step of lithography, where the rigid segments of the micro-grippers are defined on the phalanges. The thick rigid panels, which are deposited by electrodeposition techniques, consisted of a magnetic layer of nickel sandwiched between two 0.5 *μ*m layers of gold for better biocompatibility and protection of nickel from the subsequent fabrication steps involving an acid wash. Commercial electroplating solutions from Technic Inc., namely Nickel Sulfamate RTU (for nickel) and TG-25E RTU (for gold) are used for electrodeposition. Finally, a mixture of S1813 and S1805 photoresists (1:5 volume ratio) is spin coated followed by a subsequent photolithography step to define the trigger layer on the micro-grippers. The reader is directed to a previous publication [20] for a more detailed description of the fabrication protocol and the design rules adopted. The thickness of the nickel layer is set to 8.5 *μ*m for all the fabricated structures except the 100 *μ*m six-finger micro-grippers. In the latter ones, the nickel thickness is limited to 2 *μ*m due to difficulties arising from the electroplating of the magnetic layers in smaller pores.

**Fig 2:**
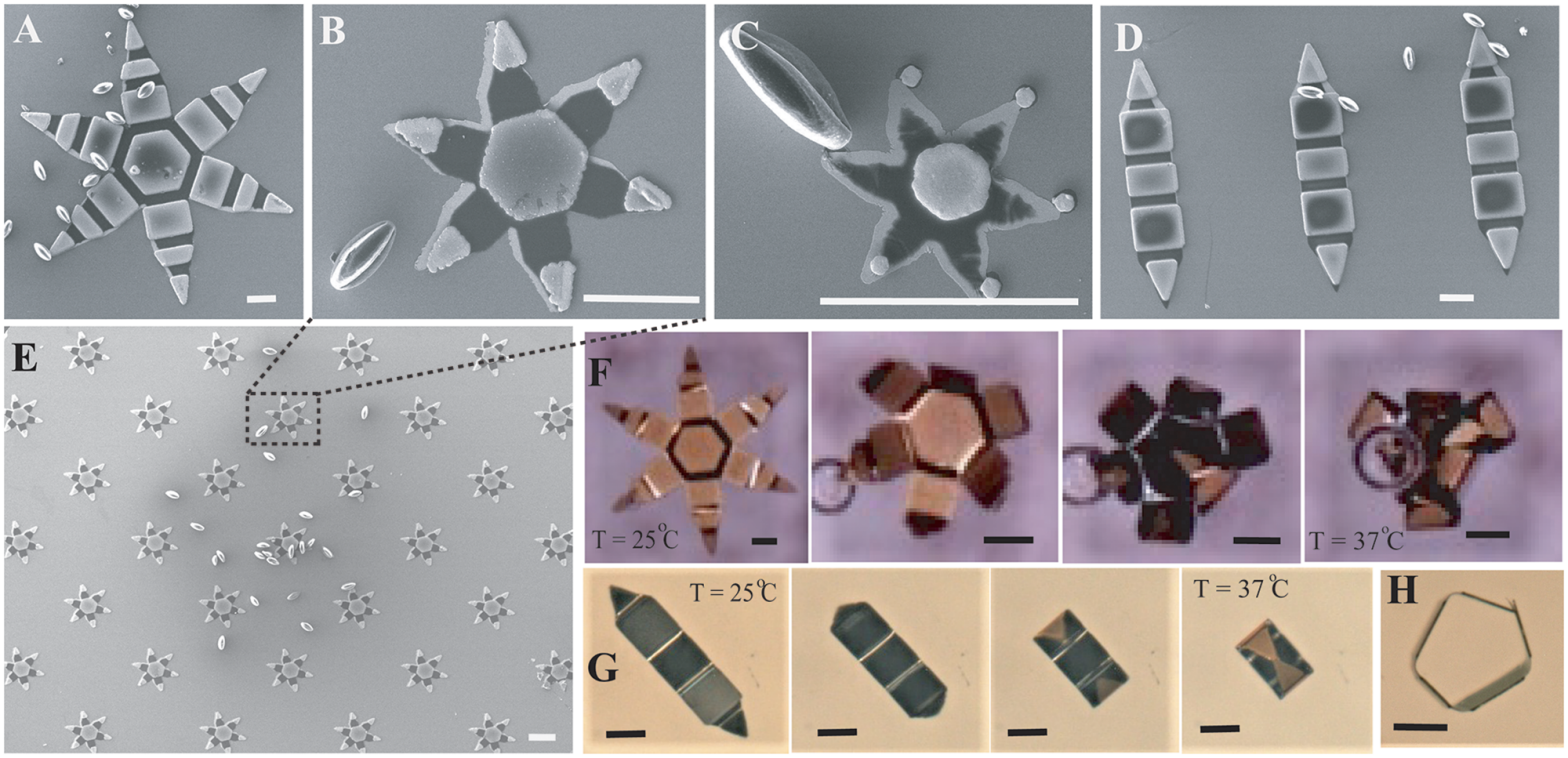
Scanning Electron Microscope (SEM) images of different shapes and sizes of micro-grippers that are used in the experiments and the size comparison with daisy pollen grains. A) 980 *μ*m six-finger; B) 250 *μ*m six-finger; C) 100 *μ*m six-finger and D) 750 *μ*m two-finger. E) SEM image showing the scalability of fabrication where a large number of micro-grippers could be simultaneously fabricated using conventional microfabrication techniques. Bright field microscopy images of thermoresponsive actuation of, a F) 980 *μ*m six-finger, and a G) 750 *μ*m two-finger micro-grippers; the grippers close as the temperature is increased from room temperature to 37°C, the physiological temperature. H) The side view of a two-finger gripper when closed. The scale bar in all the images is 100 *μ*m.

The effect of scale is analyzed by comparing three different scales (100 *μ*m, 250 *μ*m and 980 *μ*m tip-to-tip size) of a commonly used six-finger design [12, 21]. Likewise, the influence of shape is investigated introducing a two-finger shape, whose width (136.5 *μ*m) is comparable to the width of the smallest of the six-finger micro-grippers and will intuitively experience less hydrodynamic drag than a six-fingered gripper of similar size. Fig 2A–2D shows the different grippers alongside pollen grains obtained from daisy flower, which are around 60–80 *μ*m in length. When the micro-gripper is at room temperature, the trigger polymer is stiff enough to prevent the phalanges from folding. But as the temperature rises to, or above, the physiological temperature (37°C), the trigger softens and allows the folding of the bilayer at the hinges located in between the rigid parts. As can be seen from the time lapse images (Fig 2F and 2G), the micro-grippers could be actuated by changing the temperature from ambient (25°C) to the physiological temperature (37°C).

**Fig 3:**
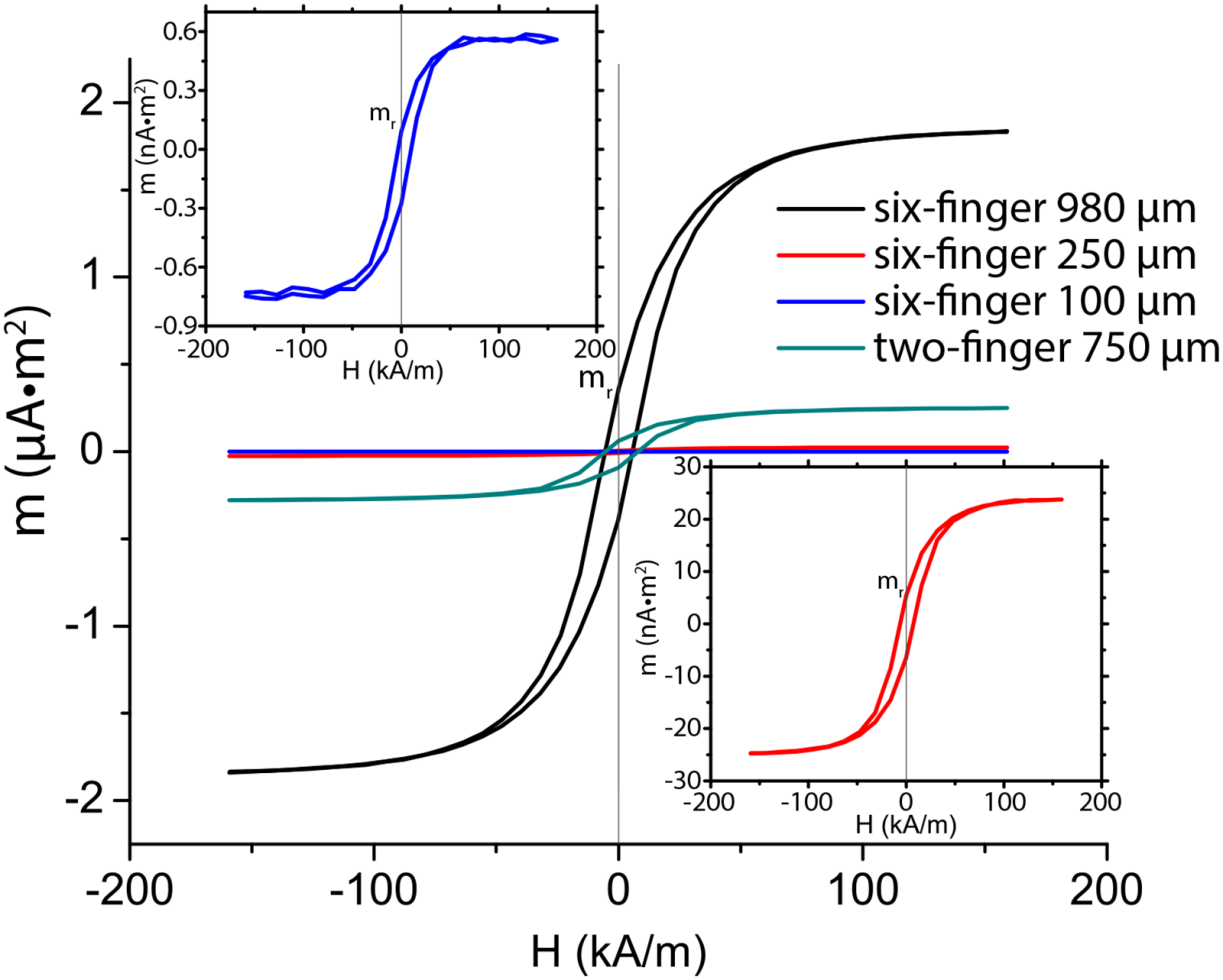
Hysteresis plots of the different designs of the four micro-grippers. The x-axis of the figure is the electromagnetic H-field, while the magnetic dipole moment (m) is shown on the y-axis. The magnetic dipole moment at remnant magnetization is marked as m_*r*_. For improved readability, the insets depict the smaller magnetic dipole moments separately.

The nickel layer on the micro-grippers imparts magnetic properties to wirelessly manipulate them using magnetic fields. A Vibrating Sample Magnetometer (GMW associates, San Carlos, USA) is used to measure the magnetic dipole moment and hysteresis of all the micro-grippers (Fig 3). The volume-dependence and the mixed (crystal and shape) magnetic anisotropy effects of the magnetic dipole moment cause it to change by three orders of magnitude, as the size of the grippers shrinks by one order of magnitude from 980 *μ*m to 100 *μ*m [22, 23]. On this regard, it should be noted that the surface area covered by the rigid segments—and therefore by nickel—doesn’t scale linearly with the overall surface area of the gripper. In point of fact, to enable thermal actuation, the relative size of the soft segments (hinges) is increased as the grippers scale down (S1 Text).

Also for reasons of shape anisotropy, we found the component of the magnetic dipole moment perpendicular to the plane of the micro-gripper to be of at least one order of magnitude smaller then the one normal to the cross section, due to the limited thickness of the micro-grippers (S1 Text). We exploit this by applying fields that are parallel to the plane of the micro-gripper. Further, in the case of the weak magnetic fields used in the current work (15 mT or 11.9 kA/m in air), the magnetic dipole moment is closer to the value corresponding to the remnant magnetization m_*r*_ (Fig 3) as mentioned in Table 1.

**Table 1:** Table summarizing the different performance and characteristics of the micro-grippers. The error on the weight measurement is obtained dividing the scale’s resolution by the number of simultaneously weighted grippers. **F**_*em*_ is used to refer to the electromagnetic force.

Gripper	Weight (*μ*N)	Estimated cross section (*μm*^2^)	Estimated surface area (*μm*^2^)	Drag coefficient	Magnetic dipole moment *(A ⋅ m^2^)*	Estimated maximum F_*em*_ *(nN)*
6-finger (980 *μ*m)	147±8	9310	2.7×105	0.04±0.01	3.9×10^−7^	23.4
6-finger (250 *μ*m)	12.3±0.4	2370	2×104	0.06±0.02	5.5×10^−9^	0.33
6-finger (100 *μ*m)	2.6±0.1	330	3.3×103	0.37±0.03	9.3×10^−11^	0.0056
2-finger (750 *μ*m)	109±3	1300	7.8×104	0.07±0.02	6.3×10^−8^	3.8

### Closed-loop control

We propose a control strategy that uses a prescribed performance approach. The control uses a combination of position and velocity errors to direct the magnetic forces (**F**_*em*_) towards a reference using an electromagnetic system [24, 25]. This system consists of an optical microscope and four orthogonally oriented iron-core electromagnetic coils (Fig 1). The coils surround a circular water reservoir (⌀40 mm) where the micro-grippers move. The prescribed electromagnetic forces are generated by applying currents to the electromagnets (further details can be found in [26]).

A Blackfly camera (FLIR, Wilsonville, USA) provides the magnified image to the custom-designed C++ tracker (described in [15]). The tracker determines the measured position of the micro-gripper. Finally, the measured position is processed by an iterative learning observer that outputs the estimated position, velocity, and acceleration of the gripper [25]. Furthermore, as the system is equipped with variable optical magnification, the micro-grippers are always controlled in a square workspace, concentric to the reservoir, of about 20 times their tip-to-tip size.

### Characterization of the micro-grippers

The translational equations of motion in the planar workspace are modeled according to:

$$\begin{array}{c} F_{em}+F_{d}+F_{i}=0, \end{array}$$ (1)

where **F**_*em*_ are the electromagnetic forces, **F**_*d*_ are the drag forces, and **F**_*i*_ are the inertial forces. Moreover, **F**_*i*_ is equal to **a**⋅*M*, where **a** is the acceleration of the gripper and *M* is its mass. The grippers move at sub-centimeter speed in a flow-less environment. Hence, **F**_*d*_ can be estimated as:

$$\begin{array}{c} F_{d}=\frac{1}{2}\rho C_{D}Av, \end{array}$$ (2)

where *ρ* is the fluid density (1,000 kg/m^3^ for water), **v** is the speed of the micro-gripper relative to the fluid, *A* is its cross sectional area normal to the direction of motion, and *C*_*D*_ is the drag coefficient. *Please refer to* Table 1 *for the numerical values of the micro-grippers characteristics*.

For characterization, the grippers are accelerated up to 1 body-length/s (*bl*/*s*), then the coils are turned off. The inertia of the micro-grippers is opposed by the viscous drag until the gripper fully stops. During the stopping motion, the grippers maintain constant orientation. Consequently, A is assumed to be constant, and their velocity can be modeled according to:

$$\begin{array}{c} v\left(t\right)=v_{o}e^{-\frac{\rho C_{D}A}{2M}\left(t-t_{o}\right)} \end{array}$$ (3)

where **v**_*o*_, *t*, and *t*_*o*_ are the initial velocity, current time, and time at which the coils were turned off, respectively. Moreover, as **F**_*em*_ and **F**_*i*_ are known, it is possible to estimate *C*_*D*_ (Table 1) by analyzing the position and velocity of the gripper over time.

Moreover, combining the magnetic dipole moment measurements (Fig 3) with previous measurements of the electromagnetic field we are able to estimate the electromagnetic force using: **F**_*em*_ = ∇(**m**⋅**B**), where **m** is the magnetic dipole moment and **B** is the magnetic flux density (Table 1) [26–28].

### Motion control in dynamic and unstructured scenario

**Fig 4:**
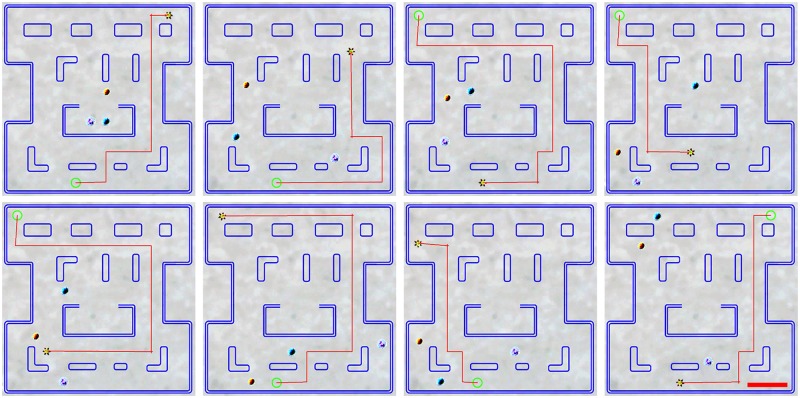
Representative trajectory of a 250 *μ*m 6-finger gripper steered towards four reference positions (green circles). The insets show representative snapshots of the controlled gripper. The red line indicates the planned trajectory of motion of the micro-gripper. The scale bar is 1 mm. *Please refer to the accompanying video that shows a representative trial of this experiment*.

Motion control experiments have been performed in a dynamic and unstructured PacMan™-like scenario (Fig 4). All the designed grippers have to fully-autonomously move inside a virtual maze from a starting position towards four pseudo-random reference positions. During their motion, they are attacked by three virtual agents (ghosts). These agents move at 1.3 bl/s and, every 5 seconds, they switch between two configured behaviors: Chase mode and Scatter mode. In Chase mode, the ghost tries to reach the micro-gripper. Some ghosts aim at the current position of the micro-gripper, others aim at a predicted future position of the miniaturized agent. In Scatter mode, the ghosts move toward the corners of the maze. To avoid these attacks the micro-grippers use a path following algorithm that iteratively computes an obstacle-free trajectory [29, 30]. In particular, the planning algorithm tries to anticipate the motion of the ghosts by estimating their future positions. In case no obstacle-free path is available, the micro-gripper is moved as far away as possible from the obstacles using an energy minimization algorithm. Finally, to grant comparability, in all the experiments the square maze is scaled to have a side of 20 body-lengths of the used micro-gripper. Fig 4 depicts a representative motion control result of a miniaturized metallic gripper. Ten experiments are performed for each of the four different designs. *Please, refer to the accompanying video that shows a representative trial of the experiment*.

### Grasping experiments

Additional experiments are performed to evaluate the grasping capabilities of these self-folding grippers. For this purpose, we release 30 samples of each of the four micro-grippers on a 5 g section of soft material (edible mozzarella cheese). Successively, the thermoresponsive hinges are actuated raising the workspace temperature above 40°C. The internal strength of the clasp is verified by first applying a weak, and then a strong magnetic field. The weak field (15 mT) is generated by the electromagnetic setup, while the strong field is generated by a cylindrical (⌀10 mm × 10 mm) N45 neodymium magnet (1.35 T) hovering at 5 mm from the water surface. However, the 6-finger 100 *μ*m micro-grippers did not produce sufficient folding angle to grasp objects (S1 Text). Hence, such grippers are excluded from this experiment.

## Discussion

The results of the characterization experiments are presented in Table 1. Here, the drag coefficient shows a strong dependence on the size of the agent. We observe that the drag coefficient increases by half, as the size decreases by 3.9 times, and by eight times as the size decreases by 9.8 times. These findings are further corroborated by the results in dynamic and unstructured scenario.

**Fig 5:**
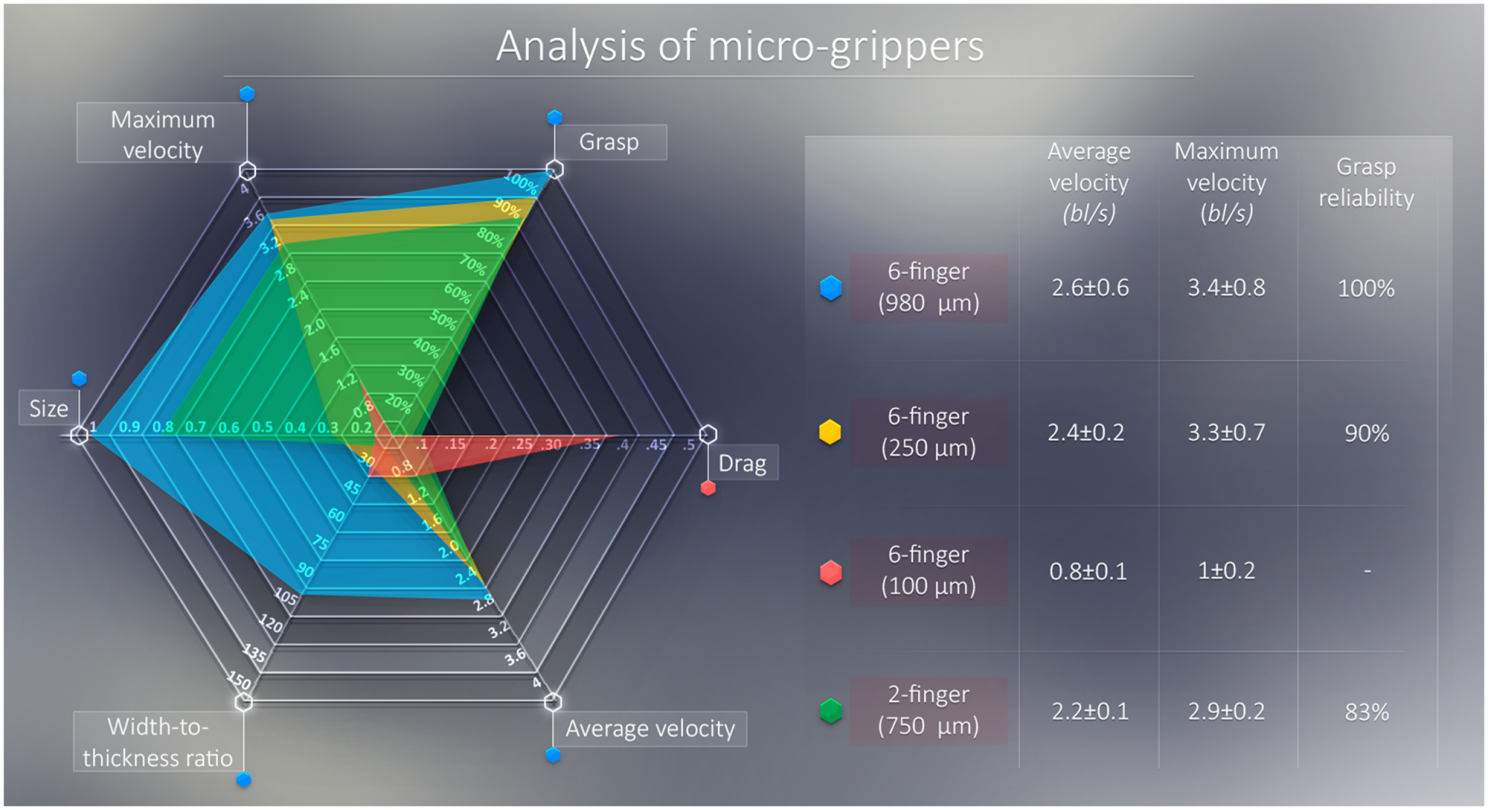
Radar graph summarizing the experimental results of the study for the four designed micro-grippers. A small colored hexagon next to the name of the gripper represents the color in the radar graph. For consistent scaling, the velocity measurements are reported in body-lengths per second (*bl*/*s*). The reliability values refer to the grasp reliability under strong fields, as no micro-gripper lost its grasp under weak fields. No grasp reliability experiments are performed with the 6-finger 100 *μ*m micro-grippers.

In point of fact, the mean and maximum velocity of the micro-grippers—which averagely traveled 216.7±30 bl per trial—is found to be in line with their drag coefficient (Fig 5). Nonetheless, it should be noted that, as the size of the gripper becomes smaller, the electromagnetic force (dependent on volume) decreases faster than the viscous friction (dependent on the cross-section; see (2)). Consequently, the maximum achievable velocity decreases as the volume of the micro-gripper decreases. An instance of this decrease can be noted comparing the 6-finger 250 *μ*m grippers to the 2-finger 750 *μ*m. While having about half the cross-section and similar drag coefficient, the 2-finger gripper is able to achieve absolute (mm/s) velocities that are three times higher than its 6-finger counterpart as a result of its higher magnetic volume. In virtue of this property, elongated shapes might be more suitable for fast navigation inside micro-channels.

Additionally, these results allow us to compute an estimate of the the theoretical force requirements to navigate these micro-grippers against blood-flow (Table 1). To this end, we assume that the micro-grippers do not experience any form of dry friction; that is, they are not in direct contact with the walls of the vessels. Further, we use an average blood density of 1055 kg/m^3^ [31] and a blood velocity of 0.3 mm/s, as it averagely is inside capillaries [32, 33]. Substituting these values in (3) we obtain a force requirement of 62 pN (980*μ*m 6-finger), 22 pN (250*μ*m 6-finger), 20 pN (100*μ*m 6-finger), and 14 pN (750*μ*m 2-finger) to maintain these grippers still. These forces are smaller than the maximum electromagnetic force (Table 1) for all but the 100*μ*m 6-finger micro-grippers. It is worth noting that, if the micro-grippers were to slide on a dry 2D substrate, the forces required for actuation would increase significantly and might even entirely impede motion. Further, the gripper would be constrained by gravity, making it only possible to grasp objects that are suspended above the agent.

**Fig 6:**
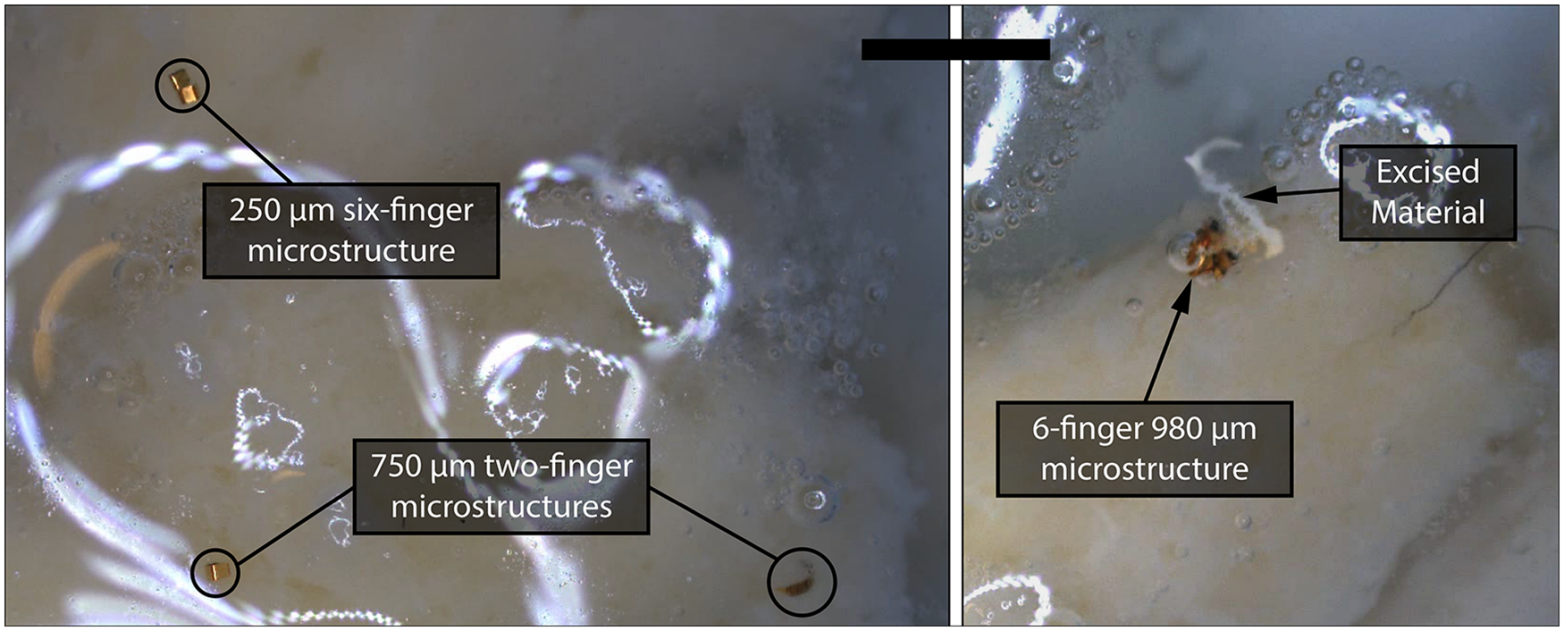
Micro-grippers during grasping experiments. *On the left*: a set of folded grippers grasping a sample of soft material (mozzarella cheese). *On the right*: a 980 *μ*m gripper after the excising a piece of mozzarella cheese. The scale bar is 1 mm. *Please refer to the accompanying video that shows a representative trial of this experiment*.

Finally, we notice how weak fields—previously shown to exert sufficient magnetic fields to move a 0.6 mg bead [15]—do not exert sufficient force/torque to dislocate or overcome the grasping force of any of the grippers. However, strong magnetic fields caused three of the 250 *μ*m 6-finger grippers and five of the 750 *μ*m two-finger grippers to lose their grasp (Fig 5). While none of the 980 *μ*m 6-finger micro-grippers released their grasp under strong fields, 46.7% of these agents and 6.67% of the 750 *μ*m two-finger excised the segment of soft material they were attached to (Fig 6). We infer this to be due to the sharp and adjacent position of the six fingers in the closed configuration, which results in the weakening—by cutting or incision—of the grasped matter, culminating in its excision when the electromagnetic force is applied. This suggests that a larger number of fingers is advantageous for tissue incision, an action required in procedures as biopsies and cytoreductions. Conversely, elongated shapes with fewer fingers are more likely to offer better dynamic performance and a more unaltering grasp, which might be more suitable for surgical interventions demanding fast micro-channel navigation—as minimally invasive endarterectomies and angioplasties, as well as targeted drug delivery.

## Conclusions

In conclusion, we design, manufacture, characterize and control four similarly-fabricated self-folding micro-grippers with different shapes and sizes. We implement a prescribed performance closed-loop control in a dynamic PacMan™-like environment. This environment presents a virtual micro-maze combined with several dynamic obstacles that attack the controlled agent along its trajectory. Not only does the developed virtual environment demonstrate the capability of all the designed self-folding metallic micro-grippers to move in unstructured environs at velocities up to 3.4 body-lengths per second, but also allows us to test their performance differences in dynamically-constrained scenarios. The obtained results also allow us to draw conclusions regarding the design criteria of such micro-grippers. Particularly, we find that tip-to-tip size and volume of the gripper are equally important as a slender shape in dynamic performance. Further, a theoretical analysis shows that three of the presented designs could theoretically be able to navigate against a blood-flow of 0.3 mm/s. Along with these techniques and demonstrations of motion control in unstructured dynamic environments, these results—while to some extent constrained to the chosen design concept—can also provide quantitative data to help designers objectively identify and exploit the aspects that are most significant for achieving the performance they desire.

In future work, we will extend this analysis and considerations to the behavior of these grippers when moving against blood-flow in a three-dimensional workspace. Additionally, the design of smaller and dexterous grippers will be investigated also considering clinically compatible imaging systems, such as ultrasound images. Finally, new methods of actuation like infrared heating or focused ultrasound will also be explored to avoid undesired heating of the environment surrounding the grippers.

## Supporting information

S1 Video Video showing representative trials of the performed experiments. (MP4)

S1 Text Supplementary information. Document presenting details regarding the constraints on miniaturization of the grippers.
(PDF)

S1 Raw Data The raw data leading to the results presented in the paper. (MAT)
