# Supplementary material for: Design, characterization and control of thermally-responsive and magnetically-actuated micro-grippers at the air-water interface
Source: PLoS One. 2017 Dec 13;12(12):e0187441. doi: 10.1371/journal.pone.0187441 (PMC5728485; doi:10.1371/journal.pone.0187441)
Supplement: S1 Text — Document presenting details regarding the constraints on miniaturization of the grippers. (PDF) [file pone.0187441.s002.pdf]

Supplementary information for

Design, comparison and control of thermal-responsive and magnetically-actuated micro-grippers on the air-water interface

Federico Ongaro[1*], Stefano Scheggi[1], Arijit Ghosh[3], Alper Denasi[1], David H. Gracias[2, 3], and Sarthak Misra[1,4]

[1]Surgical Robotics Laboratory, Department of Biomechanical Engineering, University of Twente, 7522 NB Enschede, The Netherlands. [2]Department of Materials Science and Engineering, The Johns Hopkins University, Baltimore, MD 21218, USA. [3]Department of Chemical and Biomolecular Engineering, The Johns Hopkins University, Baltimore, MD 21218, USA. [4]Department of Biomedical Engineering, University of Groningen and University Medical Centre Groningen, 9713 GZ Groningen, The Netherlands.

1. Design of the micro-grippers

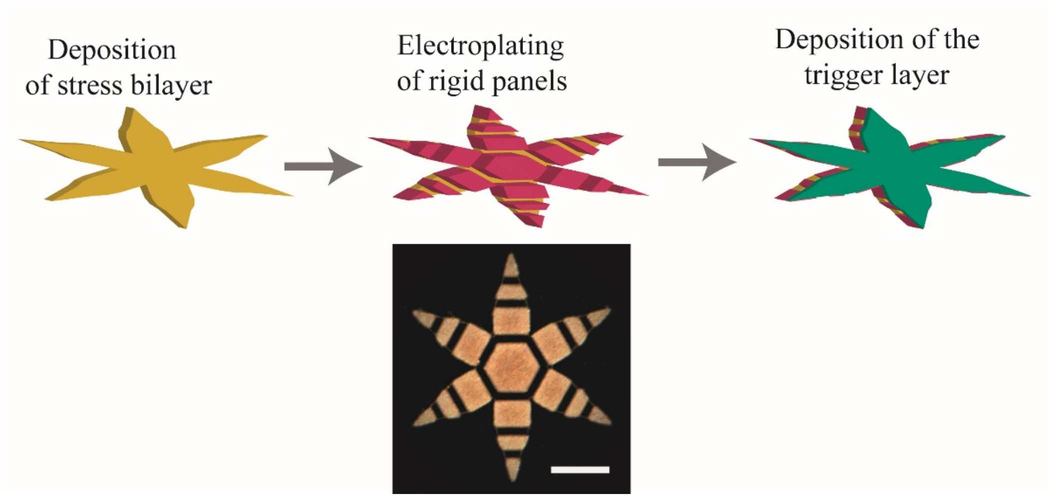

Figure S1: Schematic outline of the fabrication of the micro-grippers, showing the stress layer deposition, panel deposition and the trigger layer deposition. The image shows a 980 µm six finger gripper, with the panels lighting up. It may be noted that the magnetic nickel layer is present only on the panels that do not participate in the folding. The dark regions on each arm correspond to the hinges that result in the folding actuation. The scale bar is 200 µm.

2. Miniaturization of the grippers

The minimum size of the grippers that can be fabricated is constrained by the limitations imposed by the stress-induced folding angle. The grippers used in this work are composed of a bilayer panel of chromium and gold. There is an intrinsic stress differential present in the evaporated thin films of chromium and gold. It is this stress that controls the folding properties of the hinges in the gripper. This stress is a function of the thickness of the layer. Moreover, the final folding angle is proportional to Et^4, where E is the intrinsic

stress and *t* is the thickness of the film. Therefore, the folding angle can be estimated by using a multilayer thin film curvature model [1-2].

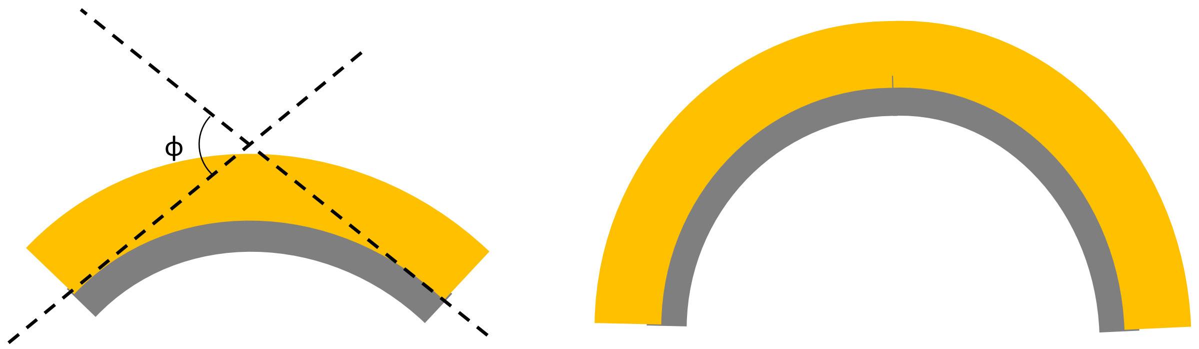

Figure S2: Folding of bilayer panels having the same thicknesses and stress, but different hinge-length. The hinge on the left is half as long as the one on the right. As shown by the figure, the panels have the same curvature. However, the difference in length yields a folding angle of about 60° for the short hinge, and of almost 180° for the longer one.

The smallest gripper presented in this work is 100 µm in length. In such gripper the hinges have a length of 27 µm. Conversely, in the 980 µm gripper, the hinges have a length of 50 µm. Additionally, the larger gripper is able to host 3 hinges per finger, while the 100 µm gripper only fits one.

The previously mentioned thin film curvature model outputs a folding angle of 126° for the 50 µm hinge and 70° for the 28 µm hinge, for panels having the same properties as the ones used in this work. The difference in the folding angle is schematically represented in Figure S2, which shows the direct correlation of the hinge length to the folding angle.

Due to these effects, the 100 µm grippers were not able to generate sufficient displacement for successful grasping of objects. The smallest gripper that could successfully achieve complete folding was found to be the 250 µm 6-finger gripper. In future work, a greater curvature – that would ensure a tighter radius - could be achieved using other materials to generate the stress mismatch [3].

3. Magnetic shape anisotropy of the grippers

The magnetic layers in the grippers have a very high aspect ratio and thus exhibit a magnetic shape anisotropy behavior [4-5], in which the easy axis of magnetization is in the plane of the grippers. This is shown in Figure S3, depicting that the in-plane magnetization (before saturation) of the 980 µm gripper is almost 10 times its out of plane magnetization.

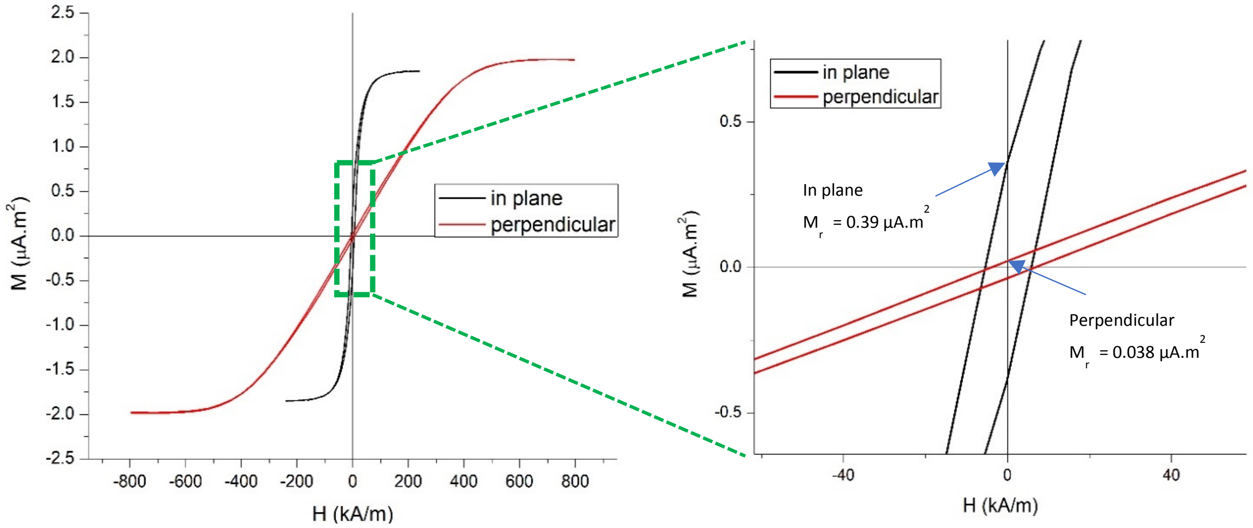

Figure S3: Magnetic hysteresis loops of the 980 μm grippers both in the in-plane and out of plane directions, showing the shape anisotropy effect of the magnetic grippers.

AFM measurements showed that the thickness of nickel layer on the 100 μm tip-tip gripper shapes is 2 ± 0.2 μm, instead of the expected 8.5 μm. Thus, while the volume of nickel present in the 980 μm grippers was around 1200 times the volume of nickel in the 100 μm grippers, the remnant magnetization of the larger grippers was around 4000 times than the smaller grippers. The 3.5 factor of difference is attributed to the higher magnetic shape anisotropy effects in the larger grippers having an aspect ratio of 12 to 30 (the smaller grippers have an aspect ratio of 5 to 14).

Gripper size	Area of nickel layer	Thickness of nickel	Volume of nickel	Remnant magnetization
980 μm	164769 μm^2	8.5 μm	1400536.5 μm^3 = 1.4×10^{-12} m^3	27.86×10^4 A/m
750 μm	54039 μm^2	8.5 μm	459331.5 μm^3= 4.593×10^{-13} m^3	13.72×10^4A/m
250 μm	8772.3 μm^2	8.5 μm	74564.55 μm^3= 7.457×10^{-14} m^3	7.376×10^4A/m
100 μm	580 μm^2	2 μm	1160μm^3 = $1.16\times10^{-15}m^3$	8.017×10^4A/m

Table showing the amount of magnetic material present in each design of the micro-grippers. We see that the remnant magnetization in the planar direction increases with the size of the grippers, due to the different aspect ratio of the nickel layer.

References

1. Nikishkov G.P., (2003) Curvature estimation for multilayer hinged structures with initial strains. *J Appl Phys.* 94: 5333–5336.

2. Leong et. al., (2009) Tetherless thermobiochemically actuated microgrippers. *PNAS*. 106(3): 703-708.
3. Malachowski et al., (2014) Self-folding single cell grippers. *Nano Letters*. 14: 4164-4170.
4. Aharoni et. al., (1998) Demagnetizing for rectangular ferromagnetic prisms. *J. Appl Phys*. 83: 3432-3434.
5. Yoo et al., (2016) Magnetic shape anisotropy effect on sensing performance and directional sensitivity in magnetostrictive nickel patch transducer. *J. Int. Mat Sys Struct.* DOI: 10.1177/1045389X15585895.
